# Different Cross-Reactivities of IgM Responses in Dengue, Zika and Tick-Borne Encephalitis Virus Infections

**DOI:** 10.3390/v13040596

**Published:** 2021-03-31

**Authors:** Karin Stiasny, Stefan Malafa, Stephan W. Aberle, Iris Medits, Georgios Tsouchnikas, Judith H. Aberle, Heidemarie Holzmann, Franz X. Heinz

**Affiliations:** Center for Virology, Medical University of Vienna, 1090 Vienna, Austria; stefan.malafa@gmail.com (S.M.); stephan.aberle@meduniwien.ac.at (S.W.A.); iris.medits@meduniwien.ac.at (I.M.); g.tsouchnikas@gmx.net (G.T.); judith.aberle@meduniwien.ac.at (J.H.A.); heidemarie.holzmann@meduniwien.ac.at (H.H.)

**Keywords:** flavivirus cross-reactivity, flavivirus IgM response, dengue, Zika, tick-borne encephalitis

## Abstract

Flaviviruses circulate worldwide and cause a number of medically relevant human diseases, such as dengue, Zika, yellow fever, and tick-borne encephalitis (TBE). Serology plays an important role in the diagnosis of flavivirus infections, but can be impeded by antigenic cross-reactivities among flaviviruses. Therefore, serological diagnosis of a recent infection can be insufficiently specific, especially in areas where flaviviruses co-circulate and/or vaccination coverage against certain flaviviruses is high. In this study, we developed a new IgM assay format, which is well suited for the specific diagnosis of TBE, Zika and dengue virus infections. In the case of TBE and Zika, the IgM response proved to be highly specific for the infecting virus. In contrast, primary dengue virus infections induced substantial amounts of cross-reactive IgM antibodies, which is most likely explained by structural peculiarities of dengue virus particles. Despite the presence of cross-reactive IgM, the standardized nature and the quantitative read-out of the assay even allowed the serotype-specific diagnosis of recent dengue virus infections in most instances.

## 1. Introduction

Flavivirus infections are of global importance for human health and pose increasing threats, as illustrated by the spread of dengue (DEN), Zika, West Nile, and tick-borne encephalitis virus (TBE) viruses (reviewed in [1,2]). These pathogens are transmitted to humans by specific arthropod vectors, either mosquitoes (dengue, Zika, West Nile, yellow fever, and Japanese encephalitis viruses) or ticks (TBE virus). The presence of the corresponding vectors and reservoir hosts determine the geographical distribution of flaviviruses, with, e.g., dengue viruses being endemic in the tropical and subtropical regions around the world and TBE virus being restricted to parts of Europe and Asia [3].

The diagnosis of flavivirus infections can be difficult, because the presence of the virus in the blood of patients (and correspondingly PCR positivity) is only transient in many instances (reviewed in [4,5]). Specific laboratory diagnosis therefore relies heavily on serological assays, and the detection of virus-specific IgM can serve as proof for a recent infection. Since all flaviviruses are antigenically related, serological analyses, however, can be complicated by cross-reactivity [6], with neutralization assays usually being most specific [4]. Further complications can arise from anamnestic immune responses and phenomena of original antigenic sin [6] that occur in the course of sequential infections in areas with co-circulating flaviviruses, but also in regions with a high degree of seropositivity due to vaccinations (TBE, yellow fever, Japanese encephalitis). The problem is therefore not unusual in travelers with pre-existing vaccine-induced immunity returning with ill-defined diseases from endemic areas. The latter situation is prominent in Central Europe, because of its high TBE vaccination coverage and many travelers returning from holiday destinations where they might have been at risk for dengue and Zika virus infections.

We assessed IgM specificity in flavivirus serodiagnosis with a newly developed IgM-capturing-ELISA format using a pre-formed detector complex with purified recombinant envelope (E) proteins of different flaviviruses. In our analyses, we performed a direct comparison of the cross-reactivity patterns in the following cohorts of recent flavivirus infections: 1. TBE patients in Austria, 2. Zika patients (travelers returning from endemic regions to Austria), and 3. dengue patients (travelers returning from endemic regions to Austria). Most of the Zika and dengue patients had a pre-existing immunity against TBE virus due to vaccination. As a control antigen for measuring broad flavivirus cross-reactivity, we used E from Rio Bravo virus (a no-known vector virus, [3]), which is distantly related to all flaviviruses included in this study.

While the IgM response was highly specific in TBE and Zika virus infections, IgM antibodies induced by dengue virus infections were substantially more cross-reactive. This discrepancy suggests differences in the structural characteristics of the three flaviviruses that affect the induction of antibodies against the most conserved sites in E. Even in the case of dengue virus infections, however, a direct quantitative comparison of IgM with all antigens in one standardized assay allowed an unambiguous differentiation between infections with these viruses, and in most instances even the identification of the infecting dengue serotype.

## 2. Materials and Methods

### 2.1. Human Serum Samples

Serum samples of patients were sent for diagnostic purposes to the Center for Virology, Medical University of Vienna, and only leftover samples were used in anonymized form. No sample was specifically collected for this retrospective study. Zika and dengue serum samples were from travelers returning from endemic regions to Austria, TBE serum samples from patients infected in Austria.

### 2.2. Dengue and Zika PCR

Viral nucleic acid was extracted from 200 µL serum using the automated NucliSens EasyMag extractor, according to the manufacturer’s instructions (Biomerieux, Marcy l´Etoile, France). Dengue and Zika RNA TaqMan-based real-time PCR assays were carried out with primers and probes located in the 3’NCR and NS5, respectively, as described previously [7,8]. Dengue serotypes were determined with the Dengue Virus Typing Tool as described [9]. The assays were validated using proficiency panels from European Centre for Disease Prevention and Control, Emerging and Vector Borne Laboratory Network (ECDC EVD-LabNet) and The Royal College of Pathologists of Australasia Quality Assurance Program and World Health Organization (RCPAQAP and WHO).

### 2.3. Serodiagnosis of TBE Cases

Serum samples were analyzed with TBE IgM and IgG ELISAs based on purified TBE virus strain Neudörfl as described previously [10]. Briefly, an IgM capture assay with horseradish peroxidase-labeled virus as detector was employed for determining IgM arbitrary units (AU) using a serum from a recent infection (set to 1000 AU) as a standard. IgG antibodies were quantified with an indirect ELISA using formalin-inactivated purified virus as antigen, and again a polyclonal post-infection TBE serum was used as a standard (set to 1000 AU).

### 2.4. Commercial Dengue IgM Kits

The DENV DetectTM IgM Capture ELISA (InBios, Seattle, WA, USA) and/or the SD Bioline Dengue Duo (Standard Diagnostics, South Korea) were used for the detection of DENV-specific IgM antibodies according to the manufacturers’ instructions.

### 2.5. Recombinant Flavivirus Antigens

The recombinant soluble E proteins (sE) were produced with the *Drosophila* Expression System (Invitrogen) using the expression vector pT389 (kindly provided by Felix Rey, Institut Pasteur), encoding the E protein lacking the stem-anchor region (synthesized by GeneArt/Thermo Fisher Scientific, Regensburg, Germany), an enterokinase cleavage site and a double strep tag (C-terminal). As described previously [11,12], Drosophila Schneider 2 (S2) cells were stably transfected and blasticidin was employed for selection. Protein expression was induced by the addition of CuSO4 and cell culture supernatants were harvested 7–10 days after induction. Recombinant proteins were purified with Streptactin-affinity chromatography (IBA GmbH, Göttingen, Germany) according to the manufacturers’ instructions. The recombinant proteins used in this study are summarized in Table 1.

### 2.6. New Flavivirus Capture IgM E-Complex ELISA

MaxiSorp microtiter plates (Nunc) were coated overnight at 4 °C with rabbit immunoglobulin directed against human µ chains (Dako) [10,11]. Serum samples in phosphate-buffered saline (PBS) pH 7.4, containing 2% sheep serum and 2% Tween 20, were added at a 1:100 dilution and incubated for 45 min at 37 °C.

For the detection of flavivirus-specific IgM antibodies, a preformed complex of strep-tagged sE and Streptactin-labeled horseradish peroxidase (HRP) was applied. sE proteins and Streptactin-HRP (IBA GmbH, Göttingen, Germany) were mixed in pre-determined optimal concentrations, incubated for 30 min at room temperature and either stored at −80 °C or directly used. The complex (final concentrations: 1 µg/mL sE, Streptactin-HRP 1:500 to 1:2000, depending on the batch) was then added to the plates and incubated for 30 min at 37 °C. Absorbance was measured at 450 nm. Each serum was tested at least twice and mean absorbance values were calculated.

The assay was validated with 38 flavivirus-negative diagnostic serum samples from previous studies [10]. The cut-off was calculated as the mean absorbance value of these negative samples plus three standard deviations. In each assay, four positive samples from confirmed TBE, Zika and dengue cases as well as at least three negative samples were included as controls.

### 2.7. Flavivirus IgG ELISA

IgG ELISAs were carried out as previously described [11]. Briefly, 25 ng/well strep-tagged sE were added to Streptactin coated microplates (IBA GmbH, Göttingen, Germany) and incubated for 1.5 h at 37 °C in PBS pH 7.4, containing 2% sheep serum and 2% Tween 20. After blocking with 1% bovine serum albumin in PBS pH 7.4, serial dilutions of samples were added and incubated for 1 h at 37 °C. Bound human antibodies were detected with HRP-labeled goat anti-human IgG (Thermo Fisher Scientific). Titers were determined by curve fitting with a four-parameter logistic regression with GraphPad Prism 8 (GraphPad Software Inc., San Diego, CA, USA). A positive control serum was used on each plate as described in [11]. The cut-off for titer calculations was determined with 32 flavivirus-negative diagnostic serum samples from previous studies and was set as the mean absorbance value from these negative controls at the 1:100 starting dilution plus three standard deviations [10,11]. Each serum was tested three times and geometric mean titers were calculated. Statistical comparisons by ANOVA were carried out with log-transformed titers and GraphPad Prism 8 (GraphPad Software Inc.). *p* values ≤ 0.05 were considered significant.

### 2.8. Flavivirus Neutralization Tests (NTs)

TBE, yellow fever (YF) and Zika NTs were performed as described previously [11,15]. Briefly, serial dilutions of serum samples were mixed with TBE virus strain Neudörfl, YF virus strain 17D or Zika virus strain H/PF 2013. BHK-21 (TBE, YF) or Vero (Zika) cells were added and incubation was continued for 3–4 days. In the case of YF and Zika viruses, virus neutralization titers were expressed as the reciprocal of the serum dilution required for protection against a virus-induced cytopathic effect (CPE). Virus neutralization titers ≥ 20 (Zika) and ≥40 (YF) were considered positive. Since TBE virus does not produce a sufficient CPE for easy read-out, virus replication and its inhibition were determined by measuring the presence of virus in the cellular supernatants with an ELISA as described previously [16,17]. The virus neutralization titer was defined as the reciprocal of the plasma sample dilution that yielded a 50% reduction in the absorbance readout compared with the control without antibody. Virus neutralization titers ≥ 10 were considered positive.

## 3. Results

### 3.1. Cohorts and Samples Analyzed in the Study

To assess the patterns of IgM cross-reactivity after different flavivirus infections, we analyzed serum samples in the novel capture IgM E-complex ELISA from three groups of patients, in which a recent flavivirus infection had been diagnosed by routine serological assays and/or direct virus detection:

1. Sixteen TBE patients (Table 2). All were found to be TBE IgM and IgG positive upon sample submission to the laboratory. Specificity of the detected antibodies was confirmed by TBE virus NTs. None of the patients had neutralizing antibodies against yellow fever (YF) virus, which would be indicative of a previous YF vaccination.
2. Twenty Zika patients (Table 3). The serum samples were obtained from travelers returning from endemic areas to Austria in 2016 and 2017. All 20 patients were shown to have Zika IgM and Zika virus neutralizing antibodies. Dengue virus infections were ruled out by testing the samples in commercial dengue IgM tests (dengue IgM negative). Zika virus infection was confirmed by PCR in 3 of the 20 patients using samples obtained 2–4 days after symptom onset (Z4, Z7 and Z20 in Table 4), when the sera were still negative in routine Zika and dengue IgM ELISAs. In these cases, follow-up samples were used for the present study.
3. Sixteen dengue patients (Table 4). The serum samples were obtained from travelers returning from endemic areas to Austria between 2009 and 2017. To identify the extent of cross-reactivity within the dengue serocomplex, we included only cases in which the infecting serotype was identified by PCR. In most instances, the first samples sent to our institution for laboratory diagnosis were PCR positive, but negative in the commercial IgM tests. The follow-up samples, however, all became dengue IgM positive (Table 4).

Consistent with the high TBE vaccination coverage in Austria, 19 of the 20 Zika patients and 15 of the 16 dengue patients had been vaccinated against TBE in the past, as confirmed by TBE NTs (Table 3 and Table 4). In addition, some TBE-vaccinated Zika (*n* = 5) and dengue (*n* = 2) patients had also been vaccinated against YF, as revealed by a positive YF NT; the single TBE-naïve dengue patient had a previous YF vaccination. The time points of vaccinations were not available to the diagnostic laboratory.

### 3.2. IgM Patterns with the E-Complex ELISA

To investigate the specificity of IgM antibodies detected in the newly developed E-complex ELISA, we analyzed the 16 TBE, 20 Zika and 16 dengue samples as displayed in Table 2, Table 3 and Table 4, respectively. This assay uses purified strep-tagged soluble E (sE) proteins of TBE, Zika, dengue serotypes 1–4 (DEN1, DEN2, DEN3, DEN4), and Rio Bravo viruses in a pre-formed detector complex with Streptactin-labeled-peroxidase in an IgM capturing format (for details see Materials and Methods).

**Figure 1:**
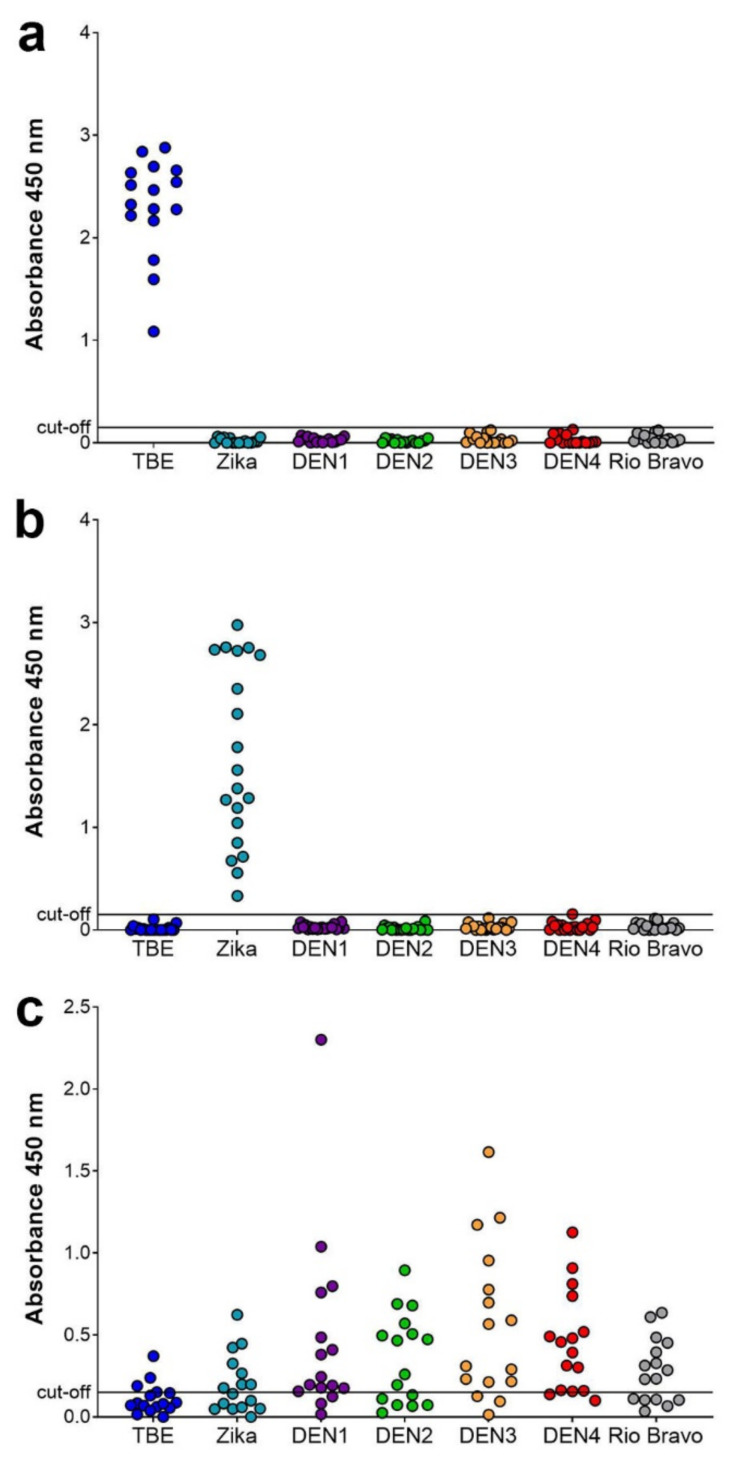
Flavivirus IgM E-complex ELISAs with the TBE, Zika and dengue serum samples. Sera from 16 TBE (**a**), 20 Zika (**b**) and 16 dengue patients (**c**) were analyzed in IgM E-complex ELISAs with soluble E proteins (sEs) of TBE, Zika, dengue (DEN)1, DEN2, DEN3, DEN4, and Rio Bravo viruses.

As displayed in Figure 1a,b, virtually no cross-reactivity was observed with the serum samples from TBE and Zika patients, showing that a highly specific serodiagnosis is possible with the assay in these cases. In contrast, a quite different pattern was obtained with the samples from dengue virus infections. These samples exhibited substantially more IgM cross-reactivity, primarily within the dengue serocomplex, but also extending to Zika, Rio Bravo and to a lesser extent to TBE viruses. Applying the cut-off as established with negative serum samples (Materials and Methods), 50% of the dengue samples yielded a positive result with Zika, 62.5% with Rio Bravo, and 20% with TBE.

**Figure 2:**
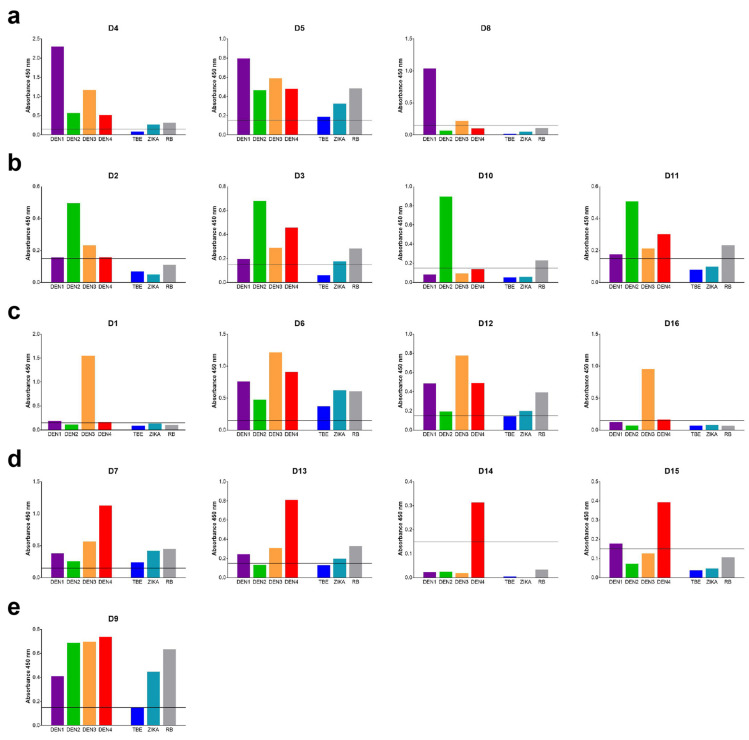
Comparative analysis of dengue serum samples in IgM E-complex ELISAs using the sE antigens of DEN1, DEN2, DEN3, DEN4, TBE, Zika, and Rio Bravo (RB) viruses. (**a**) PCR-confirmed DEN1 samples D4, D5 and D6. (**b**) PCR-confirmed DEN2 samples D2, D3, D10 and D11. (**c**) PCR-confirmed DEN3 samples D1, D6, D12 and D16. (**d**) PCR-confirmed DEN4 samples D7, D13, D14 and D15. (**e**) PCR-confirmed DEN2 sample D9 with a highly cross-reactive IgM pattern. Solid line: cut-off.

For visualizing the capacity of the assay to discriminate between infections with different dengue virus serotypes, the IgM reactivity pattern of each dengue serum with the different antigens is displayed in Figure 2. Each serum yielded an individual-specific pattern with different degrees of cross-reactivities. The comparison of reactivities with all antigens in one assay allowed the clear identification of a dengue virus infection, despite the presence of varying amounts of cross-reactive IgM to Zika, TBE, and Rio Bravo virus antigens. Quantitative evaluation even allowed a conclusion with respect to the infecting dengue virus serotype in most instances, as illustrated in Figure 2a–d. It is of note that the reactivity patterns varied strongly, even between infections with the same dengue virus serotype (serotype 1: Figure 2a, serotype 2: Figure 2b, serotype 3: Figure 2c, serotype 4: Figure 2d), suggesting strain-specific and/or individual-specific differences in inducing cross-reactive antibodies. In one serum sample (D9, Figure 2e), cross-reactive IgM antibodies dominated to such an extent (as revealed by the reactivity with the Rio Bravo control) that identification of the infecting serotype was not possible. Differentiation from Zika and TBE virus infections, however, was still unambiguous.

**Figure 3:**
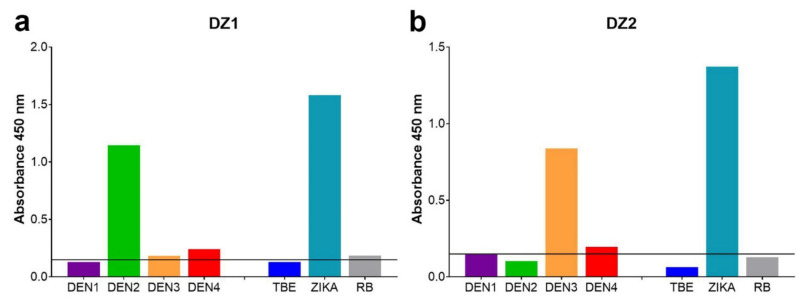
Flavivirus IgM E-complex ELISAs of two samples from suspected recent dengue and Zika virus infections with sEs of DEN1, DEN2, DEN3, DEN4, Zika, Rio Bravo (RB) and TBE viruses. (**a**) Sample DZ1; (**b**) sample DZ2. Solid line: cut-off.

Serum samples from two patients sent to our institution for dengue/Zika diagnosis yielded a pattern suggestive of a double infection with dengue and Zika viruses. Patient DZ1 (Figure 3a) had returned from a tour in Central America, patient DZ2 (Figure 3b) from Jamaica.

As shown in Figure 3, the two samples yielded a strong IgM signal with sEs from Zika and only one dengue serotype, without significant reactivity to the sEs of the other dengue serotypes, TBE and Rio Bravo viruses. Unfortunately, the samples from both patients were collected too late for virus identification by PCR.

### 3.3. IgG Patterns of Selected Dengue and Zika Patients

**Figure 4:**
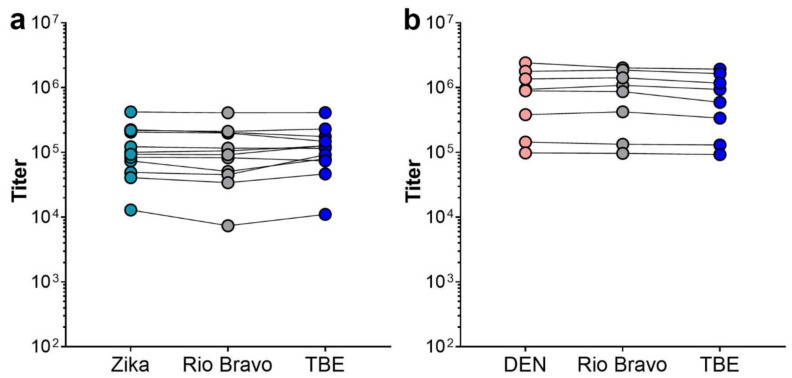
IgG ELISAs with sE antigens from different flaviviruses. Samples from 12 Zika (**a**) and 8 dengue (**b**) patients with pre-existing TBE immunity were analyzed with the sE of the corresponding infecting virus (Zika, turquoise circles in (**a**); dengue, light red circles in (**b**)) as well as Rio Bravo (grey circles) and TBE sE (blue circles). (**a**) Serum samples Z1, Z3, Z5, Z6, Z7, Z8, Z9, Z10, Z12, Z14, Z15, and Z16 from Table 3. (**b**) Serum samples D1, D2, D3, D5, D6, D7, D9, and D12 from Table 4. ANOVA revealed no significant differences between IgG titers obtained with the homologous and heterologous antigens.

Since most of the Zika and dengue patients had previous flavivirus vaccinations (Table 3 and Table 4), we also analyzed the cross-reactivity patterns of IgG antibodies of a subset of 12 Zika and 8 dengue patients. The selection was based on the availability of sufficient sample volume. We determined IgG titers by ELISAs with the sE antigen corresponding to the infecting virus (Zika or the identified dengue serotype), as well as Rio Bravo and TBE sE. As shown in Figure 4, each of the samples yielded virtually the same titer with the different antigens, indicating the dominance of broadly flavivirus cross-reactive IgG antibodies in these sera.

## 4. Discussion

**Figure 5:**
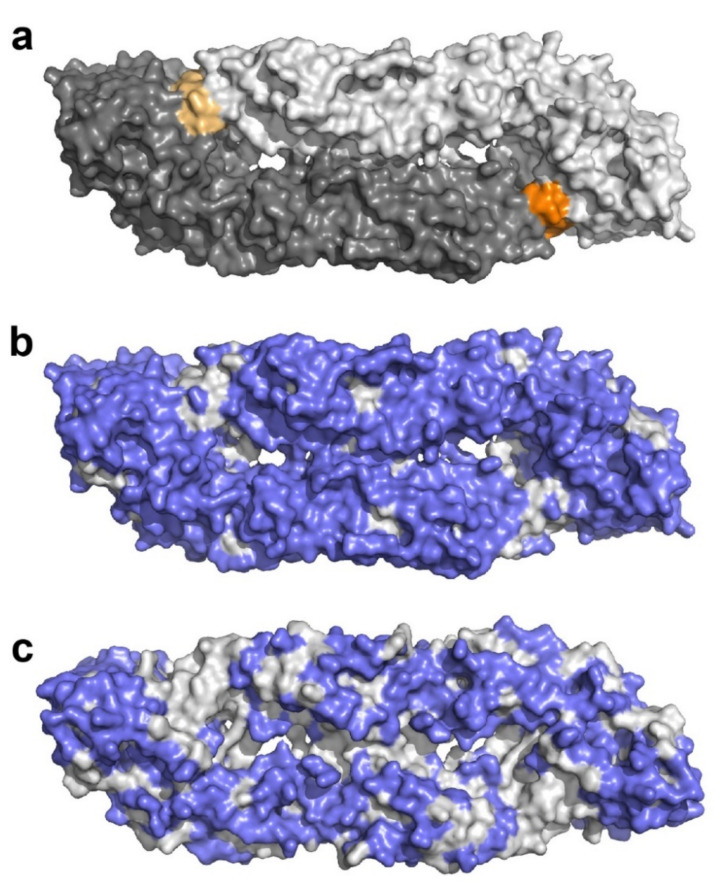
Surface representation of the Zika virus sE dimer in a top view. (**a**) The two monomers are shown in light and dark gray, the highly conserved fusion loops in light and dark orange, respectively. (**b**) Surface-exposed residues that differ between DEN1-4, Zika, Rio Bravo and TBE viruses highlighted in blue. (**c**) Surface-exposed residues that differ between DEN1-4 viruses highlighted in blue. Amino acid sequences of E proteins of the different flaviviruses listed in Table 1 were aligned with MUSCLE (https://www.ebi.ac.uk/Tools/msa/muscle/, accessed on 16 February 2021). The image was prepared with PyMol using the Protein data bank (PDB) files 6CO8 [20] and 1OAN [21].

In this study, we show that a newly developed E-complex capture IgM assay allows a highly specific serodiagnosis of flavivirus infections. The response was type-specific for the infecting virus in the case of TBE and Zika virus infections, without detectable broadly flavivirus cross-reactive IgM antibodies. In contrast, substantial amounts of such IgM antibodies were found in dengue virus infections, as determined by their reactivity with the distantly related Rio Bravo virus E protein (Figure 1 and Figure 2). These results indicate substantial differences in the presentation of conserved epitopes in courses of infection with dengue, Zika and TBE viruses. Broadly flavivirus cross-reactive antibodies mainly target a highly conserved region in the E protein, the so-called fusion loop, which is buried in the homodimeric structure of E at the dimer interface (Figure 5) (reviewed in [18]). A number of studies have shown that this region can become more exposed by dynamic motions of the E proteins on the virus surface (“viral breathing”) and is also presented in partially immature virions [18,19]. Our data suggest that in the course of dengue virus infections the fusions loops might be more exposed, and therefore higher amounts of broadly cross-reactive antibodies are induced than during infections with Zika and TBE viruses.

Despite the induction of substantial amounts of cross-reactive IgM antibodies in dengue virus infections, differentiation from Zika virus infections (co-circulating in the regions from which the travelers returned) was possible in all cases analyzed (Figure 2). As expected from the closer sequence and antigenic relationship, the extent of cross-reactivity was higher within the dengue serocomplex than in other flaviviruses. The direct and parallel quantitative comparison of reactivities with all four dengue virus antigens, however, even allowed determination of the infecting serotype in 15 out of 16 cases (Figure 2). The high specificity of the assay also made identification of a potential double infection with Zika and dengue viruses possible (Figure 3). Such double infections might occur in regions where several flaviviruses co-circulate and/or through travel activities to regions endemic for different flaviviruses. Indeed, both of the two patients with the dengue/Zika pattern had a corresponding travel history to Central America and the Caribbean, respectively (Figure 3).

Notably, the patterns of IgM cross-reactivity after dengue virus infections are quite divergent and apparently subject to strong individual variation (Figure 2). Some patients developed mostly IgM antibodies specific for the infecting dengue serotype (e.g., Figure 2a sample D6, Figure 2b sample D10, Figure 2c sample D1) with no detectable cross-reactivity, similar to what was seen in Zika and TBE patients (Figure 1a,b); others developed more and different specificities of cross-reactive antibodies (e.g., Figure 2a sample D5, Figure 2e). Detailed molecular studies have shown that even small structural changes such as point mutations in E can strongly affect the heterogeneity of dengue virus particles and their breathing behavior [22]. The IgM reactivity patterns observed might therefore be a result of infections with different dengue virus strains that exhibit varying degrees of fusion loop exposure. Considering the travel history of the dengue patients to destinations where dengue serotypes co-circulate, we cannot rule out effects of multiple infections on IgM cross-reactivity patterns. However, these are less likely to occur in short-time travelers than in people living permanently in these areas.

Dengue serotyping by IgM assays was also reported in previous studies, and it was found to be more reliable in the case of primary than secondary dengue virus infections [23,24,25,26,27]. Our serum samples were from Austrian travelers, and thus they all were most likely primary dengue virus infections. Of the 16 cases, only one could not be serotyped, but this patient also had the highest amount of broadly flavivirus cross-reactive IgM antibodies, as revealed by the reactivity with Rio Bravo virus E (Figure 2e). Future studies should therefore address analysis of IgM patterns in sequential samples from primary and secondary dengue virus infections. The induction of high amounts of broadly cross-reactive antibodies might be induced by infecting strains with a strong propensity of breathing and/or particle heterogeneity [18,19], favored by co- or sequential infections with different dengue virus strains in endemic regions [28]. Several studies suggest a diversity in the memory B-cell population, showing that these cells cannot only express IgG, but also IgM antibodies [29,30]. IgM memory might thus also play a role and contribute to a lower reliability of serotyping in secondary dengue virus infections.

The importance of serological assays for the correct diagnosis of flavivirus infections, in particular IgM tests, is underlined by the fact that molecular techniques of virus detection in the blood are limited to the acute viremic phase, which is often missed at the time of first analysis [5]. In the case of TBE virus, neurological symptoms occur two to three weeks after infection, and this is the time point when people usually seek medical advice. Therefore, PCR plays virtually no role in the laboratory diagnosis of TBE (Table S1). Detection of viral RNA is more important for the diagnosis of dengue and Zika virus infections. However, travelers returning from endemic regions to Central Europe contact physicians relatively late, mostly when symptoms worsen, and PCR very often already yields negative results in these cases. Consistent with this notion, only ~11% and 23% of the Zika and dengue cases were diagnosed by PCR at our institution (Table S1). This situation underlines the importance of specific serological assays. Especially in the case of dengue virus infections, the presence of broadly cross-reactive IgM antibodies may lead to false interpretations, unless a comprehensive and quantitative analysis is performed with relevant flaviviruses.

## Figures and Tables

**Table 1:** Recombinant soluble E (sE) proteins used as antigens in ELISAs.

Recombinant sE Protein	Amino Acids (E)	Virus Strain	Genbank	Reference
DENV serotype 1	1–399	FGA/89	AAF82039	This study
DENV serotype 2	1–399	16681	NC_001474	[11,12]
DENV serotype 3	1–397	CH53489	DQ863638	This study
DENV serotype 4	1–399	814669	NC_002640	This study
Zika virus	1–408	H/PF/2013	KJ776791	[13]
Rio Bravo virus	1–393	RiMAR	AF144692	[11,12]
TBEV sE	1–400	Neudoerfl	U27495	[14]

**Table 2:** Characteristics of serum samples obtained from tick-borne encephalitis (TBE) patients in Austria, 2017, as determined by routine serodiagnosis.

Sample	TBE IgM ^1^	TBE IgG ^1^	TBE NT ^2^	YF NT ^3^
T1	>1000	>1000	pos	neg
T2	900	>1000	pos	neg
T3	567	>1000	pos	neg
T4	664	>1000	pos	neg
T5	>1000	>1000	pos	neg
T6	>1000	>1000	pos	neg
T7	>1000	>1000	pos	neg
T8	968	>1000	pos	neg
T9	995	>1000	pos	neg
T10	>1000	>1000	pos	neg
T11	>1000	>1000	pos	neg
T12	>1000	>1000	pos	neg
T13	>1000	>1000	pos	neg
T14	>1000	>1000	pos	neg
T15	>1000	>1000	pos	neg
T16	829	>1000	pos	neg

^1^ TBE virus-specific IgM and IgG were quantified in arbitrary units as described in Materials and Methods. All samples were IgM and IgG positive; ^2^ TBE neutralization test; ^3^ Yellow fever (YF) neutralization test.

**Table 3:** Characteristics of Zika serum samples obtained from travelers who returned to Austria from Zika-endemic regions, 2016–2017, as determined by routine diagnosis.

Sample	Zika IgM	DEN IgM ^1^	Zika NT	TBE NT	YF NT	Infection Site
Z1	pos	neg	pos	pos	neg	Brazil
Z2	pos	neg	pos	pos	neg	Columbia
Z3	pos	neg	pos	pos	pos	Columbia
Z4	pos	neg	pos	neg	neg	Brazil
Z5	pos	neg	pos	pos	neg	Columbia
Z6	pos	neg	pos	pos	pos	Venezuela
Z7	pos	neg	pos	pos	neg	Dominican Republic
Z8	pos	neg	pos	pos	neg	Dominican Republic
Z9	pos	neg	pos	pos	neg	Dominican Republic
Z10	pos	neg	pos	pos	neg	Mexico
Z11	pos	neg	pos	pos	n.a.	Tobago
Z12	pos	neg	pos	pos	neg	Mexico
Z13	pos	neg	pos	pos	neg	Nicaragua
Z14	pos	neg	pos	pos	neg	Barbados
Z15	pos	neg	pos	pos	neg	Netherlands Antilles
Z16	pos	neg	pos	pos	pos	Netherlands Antilles
Z17	pos	neg	pos	pos	neg	Cuba
Z18	pos	neg	pos	pos	pos	Cuba
Z19	pos	neg	pos	pos	pos	Columbia
Z20	pos	neg	pos	pos	neg	Vietnam

^1^ Dengue (DEN) virus-specific IgM were detected with commercial kits as described in Materials and Methods.

**Table 4:** Characteristics of dengue serum samples obtained from travelers who returned to Austria from dengue-endemic regions, 2009–2017, as determined by routine diagnosis.

Sample	DEN PCR ^1^	DEN IgM ^2^	TBE NT	YF NT	Infection Site
D1	Den3	pos	pos	neg	Bali
D2	Den2	pos	pos	neg	Brazil
D3	Den2	pos	pos	pos	Unknown ^3^
D4	Den1	pos	pos	neg	Thailand
D5	Den1	pos	pos	neg	Madeira
D6	Den3	pos	pos	neg	Unknown ^3^
D7	Den4	pos	pos	neg	Thailand
D8	Den1	pos	pos	neg	Thailand
D9	Den2	pos	neg	pos	Tanzania
D10	Den2	pos	pos	neg	Maldives
D11	Den2	pos	pos	neg	Sri Lanka
D12	Den3	pos	pos	neg	Indonesia
D13	Den4	pos	pos	pos	Philippines
D14	Den4	pos	pos	neg	Philippines
D15	Den4	pos	pos	neg	Sri Lanka
D16	Den3	pos	pos	neg	India

^1^ 1st samples used for confirmation by PCR; ^2^ dengue virus-specific IgM were detected with commercial kits as described in Materials and Methods; ^3^ specific travel history not available to the diagnostic laboratory.

## Data Availability

Data are contained within the article or supplementary material.

## Supplementary Materials

The following are available online at https://www.mdpi.com/article/10.3390/v13040596/s1, Table S1: Molecular diagnosis of TBE, Zika and dengue at the Center for Virology, Medical University of Vienna, Austria, 2016–2019.
