# Supplementary material for: Different Cross-Reactivities of IgM Responses in Dengue, Zika and Tick-Borne Encephalitis Virus Infections
Source: Viruses. 2021 Mar 31;13(4):596. doi: 10.3390/v13040596 (PMC8066087; doi:10.3390/v13040596)
Supplement: Supplementary file 1 [file viruses-13-00596-s001.pdf]

Table S1. Molecular diagnosis by PCR of TBE, Zika and dengue at the Center for Virology, Medical University of Vienna, Austria, 2016–2019.

	Number of Cases		
	**PCR pos**	**Total**	**% pos**
**TBE**			
2016	0	89	
2017	0	116	
2018	1	154	
2019	1	108	
**2016-19**	**2**	**467**	**0.43**
**Zika**			
2016	3	39	
2017	1	7	
2018	0	0	
2019	1	1	
**2016-19**	**5**	**47**	**10.64**
**Dengue**			
2016	18	105	
2017	18	70	
2018	13	57	
2019	32	116	
**2016-19**	**81**	**348**	**23.28**
